# Firm innovation activities and consumer brand loyalty: A path to business sustainability in Asia

**DOI:** 10.3389/fpsyg.2022.942048

**Published:** 2022-07-25

**Authors:** Lin Yi, Muhammad Saqib Khan, Asif Ali Safeer

**Affiliations:** ^1^School of Physical Education, Huazhong University of Science and Technology, Wuhan, China; ^2^School of Management, Huazhong University of Science and Technology, Wuhan, China; ^3^Business School, Huanggang Normal University, Huanggang, China

**Keywords:** innovation activities, brand loyalty, brand preference, brand prototype, brand recommendation

## Abstract

**Background:**

In recent years, technological advancements have increased the importance of innovation activities. Therefore, firms invest millions of dollars in innovation activities to ensure long-term business sustainability. Similarly, consumer concerns have increased dramatically over the past years. Thus, brand loyalty has become a top priority for firms and consumers. In this background, this research examines how firms’ innovation activities translate into consumer brand loyalty to assure business sustainability in Asian markets, particularly China, Pakistan, and Indonesia.

**Objectives:**

This study’s specific objectives are to comprehend the concept of firms’ innovation activities and their effect on the brand prototype. Examine the effect of the brand prototype on global brand preference, recommendation, and loyalty among Asian consumers. Find out the impact of brand preference on brand recommendations and the influence of brand recommendations on brand loyalty among Asian consumers.

**Materials and methods:**

A total of 814 consumers from Asian countries (China, Pakistan, and Indonesia) participated in this study, and structural equation modeling was used to analyze the data.

**Results:**

The findings indicate that firms’ innovation activities, such as processes, products, and store environment, positively influenced the brand prototype, thereby increasing consumer brand knowledge. Likewise, brand prototype contributes to developing brand preference, brand recommendation, and brand loyalty among Asian consumers. Lastly, consumer brand preference significantly influenced brand recommendation, which positively improves consumer brand loyalty in Asia.

**Conclusion:**

This study concluded that Asian (Chinese, Pakistani, and Indonesian) consumers have favorable perceptions of firms’ innovation activities (i.e., process, product, and store environment innovation), which influences their ability to develop brand prototypes to increase consumer brand knowledge. Similarly, brand prototype fosters brand preference, recommendation, and loyalty. Likewise, favorable brand preference encourages consumers to recommend the brand to others, strengthening brand loyalty. Thus, firms should invest in innovation activities to strengthen consumer brand loyalty in Asian markets. Consequently, this study may assist multinational corporations in increasing their business volumes and market shares in Asia.

**Managerial recommendations:**

This study provides important managerial recommendations. The findings revealed that global managers can develop and implement several branding strategies for sustaining their businesses in the Asian environment.

## Introduction

With the rapid pace of technology development, the impact of innovation on a business’s success has garnered great academic and managerial attention (Purchase and Volery, 2020; Breitling and Scholl, 2022; Hu et al., 2022; Wang et al., 2022). Similarly, innovation is essential for organizations to create competitive advantages over competitors (Saqib and Satar, 2021). Thus, many global firms invest millions of dollars annually in innovation and research and development activities (Li et al., 2021). Despite technological developments, many innovations fail, while few firms successfully market innovations that contribute to revenue and growth. Thus, the success of any innovation is contingent on how consumers perceive and respond to it, whether positively or negatively (Chiesa and Frattini, 2011). Planning innovation activities in a consumer-centric framework is a significant challenge, as many corporations rely on expert advice while ignoring the consumer perspective (Kunz et al., 2011). As a result, such innovation may be doomed to fail miserably because today consumers have many choices and are more empowered than ever before (Fitzgerald et al., 2020). In this situation, global corporations must change their thinking pattern by assessing their products, processes, store environment, and marketing strategies through consumers’ lenses in order to sustain their businesses and remain competitive in the global markets (OECD, 2018; Melluso et al., 2020).

Today, brands are essential components of innovation that assist in launching new products and services. They foster innovation ownership, credibility, and acceptability, enhance visibility and facilitate communication (Purchase and Volery, 2020). Prior research revealed that brand performance plays a substantial role in innovation performance (Sharma et al., 2016). Thus, consumer perceptions of innovation may increase brand loyalty (Lin et al., 2019). Therefore, global firms are increasingly concerned about their brands because of changing consumer behavior in branding (Safeer et al., 2021b). Thus, they modify their innovative operations to achieve long-term sustainable growth by enhancing consumer brand loyalty (Loučanová et al., 2021). Consumer acceptance of new products and services offered by firms is critical to the success of any innovation. Thus, the innovation performance is determined by the consumer response to new products. As a result, consumer responses affect firms’ innovation activities (Lowe and Alpert, 2015). Therefore, firms must revamp their products through innovation, pay attention to their processes and store environment and implement innovative marketing strategies to influence consumers in global markets (Zameer et al., 2019). Previous research revealed that the development of brand preference indicates consumer responsiveness to business innovation (Chowdhury and Khare, 2011). Similarly, understanding consumer preferences supports the development of successful innovation initiatives for corporations. Thus, successful innovation initiatives contribute to the retention of existing and the acquisition of new consumers (Liu and Atuahene-Gima, 2018).

Previous research has focused primarily on innovation activities in the organizational setting and measured innovation from several perspectives. For example, many authors discovered that marketing innovation increases organizational competitiveness in India (Gupta et al., 2016), SMEs’ sustainable competitive advantage in Ghana (Quaye and Mensah, 2019), and firms’ exports in the Spanish context (Medrano and Olarte-Pascual, 2016). Other authors discovered that product and marketing innovation boosts market performance in Turkey (Aksoy, 2017) and positively impacts sustainable competitive advantage and performance in South Korea (Na et al., 2019). Similarly, product, process, and marketing innovation positively impact the performance of the knitting industry in the Brazilian context (Ganzer et al., 2017). However, few studies have examined the impact of innovation on consumer behavior from a branding standpoint by employing different concepts. For instance, previous research examined innovation activities in Asian and European contexts, including brand equity in Denmark (Nørskov et al., 2015), satisfaction and word of mouth in Spain (Fuentes-Blasco et al., 2017), brand recommendation and willingness to pay in China (Zameer et al., 2019; Zhang et al., 2020). Thus, it demonstrates that few research studies with a narrow scope have been undertaken in Asia. In contrast, brand loyalty among consumers is a serious issue in the present era (Safeer et al., 2021c), and several researchers advocate for additional research on consumer brand loyalty by examining firms’ innovation activities through the lens of consumer perceptions (Zameer et al., 2019; Ertemel et al., 2021; Safeer et al., 2021c). As a result, this research proposes that “how consumers perceive firms’ innovation activities affect their brand loyalty via brand prototypes, preferences, and recommendations?” Therefore, this study pursues the following specific aims in the context of Asia, including China, Pakistan, and Indonesia:

To understand the concept of firms’ innovation activities and their impact on the brand prototype.

To understand the concept of brand prototype and its impact on consumers’ brand preference, recommendation, and loyalty.

To ascertain the impact of brand preference on brand recommendations and the effects of recommendations on consumer brand loyalty.

Using categorization theory, this research contributes three-fold. First, this research explains the different types of firms’ innovation activities and examines their effects on the brand prototype, which may be helpful in enhancing the consumers’ knowledge about global brands. Second, this research elucidates the effects of the brand prototype (knowledge) on brand preference, recommendation, and loyalty, which may assist the managers in revisiting their branding strategies to retain existing and acquire new consumers in Asian markets. Third, this study examines the influence of consumer brand preference on consumers’ brand recommendations to other consumers, as well as the effect of brand recommendations on consumer brand loyalty. Therefore, understanding consumer behavior may benefit global managers in fostering brand loyalty among consumers to sustain long-term business in Asia.

To begin structuring this research, we discuss the significance of this topic, the gap, and the research objectives. Then we define the comprehensive literature review, development of hypotheses, methods, results interpretation, and discussion of findings in light of prior research. Finally, we conclude this research with theoretical and managerial implications, as well as its limitations and future research agenda.

## Literature review

### Perceived process innovation

Perceived process innovation is “the implementation of a new or significantly improved production or delivery method. It includes significant changes in techniques, equipment or software” (Bloch, 2007). Manual (2005) defines process innovation as organizations’ new product development using new production processes or technologies. Kahn (2018) emphasized that process innovation is the prime objective of managers, which helps them to boost organizational effectiveness by increasing product performance and lowering costs as well as coping with market competition. Further, disseminating information about process innovation through the media or the company’s own channels enables consumers to create perceptions of the firm’s process innovation (Lee and Kim, 2013). Previous research has examined process innovation in different environments. For example, Tolentino (2017) evaluated organizations’ process innovation based on their commitment and usage of new technology for research and development. Ganzer et al. (2017) demonstrated that process innovation develops and improves the textile products manufactured and delivered in Brazil. Elia et al. (2020) stated that a virtual brand community observed through the lens of process innovation can be employed as a tool for open innovation in the semiconductor sector.

### Perceived product innovation

Perceived product innovation is “a new or improved good or service that differs significantly from the firm’s previous goods or services, and that has been introduced on the market” (OECD, 2018). According to Kahn (2018), product innovation refers to introducing new products or services to the market. Various types of product innovation are available since innovation extends from incremental to radical offers (Geng et al., 2021). This is perceived as product innovation when new products are continuously introduced to the market (Kim et al., 2013). Previous research contributed to product innovation in a different context. For instance, Nørskov et al. (2015) discovered that product innovation attributes contributed significantly to brand equity in the Danish market. However, the effects of high vs. low brand equity may vary. They observed that low equity brands benefited more (of product attributes) than high equity brands. Aksoy (2017) discovered that product innovation boosts the market performance of small and medium enterprises (SMEs) in the Turkish environment. Markovic and Bagherzadeh (2018) analyzed data from 1,516 Spanish firms and discovered that product innovation significantly increases the innovation performance of Spanish firms. Ardani (2019) discovered that Unilever’s product innovation greatly influences the purchasing decisions of Indonesian customers. Caniago (2020) revealed that product innovation directly improves the firm’s performance, emphasizing that a greater benefit of product innovation would increase the value of firms. Hu et al. (2022) stated that a close product distance assists in sustaining current comparative advantages and encourages product innovation by acquiring potential comparative advantages to increase the variety of medical device products in Asia.

### Perceived marketing innovation

Perceived marketing innovation strives to establish new and various sorts of connections with consumers and may include new promotional initiatives. Thus, marketing innovation contributes to demand generation by increasing awareness, brand familiarity, and product distinctiveness (Kahn, 2018). The brand’s marketing innovation is perceived through its promotional campaign, channel marketing, and other advertising strategies. Marketing innovation incorporates 4Ps, i.e., product, price, placement, promotion, plus marketing information system (Gupta et al., 2016). Despite the enormous progress in innovation research, distinctive marketing strategies have received little attention (Moliner-Velázquez et al., 2019; Branstad and Solem, 2020). Various recommendations have been recently made to examine marketing innovations that result from novel approaches to designing, pricing, promoting, and distributing products or services (Grimpe et al., 2017; Narayanan and Das, 2021). The plethora of existing concepts and the ambiguities of how marketing innovation conceptions are operationalized have caused some misunderstanding when directly comparing empirical studies, requiring that academics define the nomenclature. Likewise, marketing managers frequently find it hard to comprehend the essence of marketing innovation, as well as its causes and effects (Purchase and Volery, 2020). Previous research examined marketing innovation in different environments. For example, Fuentes-Blasco et al. (2017) discovered that marketing innovation improves the store image and consumer value in Spanish settings. Similarly, other research studies discovered that marketing innovation enhances the competitiveness of SMEs in European corporate culture (Ungerman et al., 2018), sustainable competitive advantages in South Korea (Na et al., 2019), and increases the value of all associated stakeholders in Indian context (Narayanan and Das, 2021).

### Perceived store environment

The perceived store environment is “the design of a retail store that effectively communicates the brand’s value to consumers, delivers brand experience, and directs consumers around the store efficiently” (Kumar and Kim, 2014). Prior research demonstrated that organizations store market-related actions as part of their firm’s product, process, and marketing strategy, which express innovation-related information (Frank et al., 2016). Chuchu et al. (2018) discovered that an innovative store environment significantly enhances the brand experience and brand attitude in the South African market. Similarly, an innovative store environment significantly improves consumer brand knowledge in China (Zameer et al., 2019). In the modern era, consumers are targeted with a wide range of products, services, and related information through brand visibility (Shao et al., 2019), brand marketing, and store environment (Kumar and Kim, 2014) in various aspects. Similarly, consumers utilize information and create a brand prototype for product evaluation based on their experiences and expectations. Thus, it is revealed that consumers’ perceptions might vary due to changing firms’ (store environment) innovation activities, which may affect brand prototypes (Loken et al., 2008).

### Brand prototype

The brand prototype is directly associated with consumer brand knowledge (Keller, 1993). Other authors (Lianxiong and Huihuang, 2010) discussed that the brand prototype is the consumer’s perceptions of the universality of brands; it incorporates the consumer’s fundamental knowledge and criteria for the brand. The brand prototype serves as a building block in the organization of brand knowledge, with customers continuing to categorize the brand and using it as a purchasing decision tool (Cao et al., 2017). Past research revealed that consumers build brand prototypes according to their backgrounds, experiences, expectations, and interpretations of the link between cognitive objects (Sun et al., 2017). Brand prototype improves the consumer’s knowledge of brands and may positively influence consumer preference, endorsement, and product sophistication (Zameer et al., 2019).

### Brand preference

Brand preference refers to a consumer’s proclivity to use a specific brand’s product over a competitor’s (Stach, 2017). Prior research has demonstrated the importance of brand preference and its impact in different consumer settings (Feng et al., 2019; Ma et al., 2019). For example, Ebrahim et al. (2016) discovered that consumer brand preferences increase mobile phone repurchase intentions among Egyptian consumers. Similarly, brand preference positively impacted consumers’ word of mouth, which increased the likelihood that they would recommend the brands to other consumers in Iran (Jalilvand et al., 2016). Prior research primarily examined the brand preference on purchase/repurchase intentions (Ebrahim et al., 2016; Maymand and Razmi, 2017; Dam, 2020). Some authors argued that consumer brand preference might significantly influence a brand’s recommendations (Zameer et al., 2019). Thus, brand preference has been an essential construct in recent years.

### Brand recommendation

The brand recommendation is the process of using positive reviews of consumers to influence other consumers (Martínez Cevallos et al., 2020). Many businesses are concerned about consumer brand loyalty in today’s uncertain environment. As a result, favorable consumer word of mouth and brand recommendations may impact consumer brand loyalty (Gounaris and Stathakopoulos, 2004). Bıçakcıoğlu et al. (2018) demonstrated that brand recommendations are the most anticipated behavioral outcomes that are favorably associated with consumer brand loyalty. In addition, other research studies have asserted that consumers’ brand recommendation is a component of brand loyalty that would positively influence consumer behavior in the tourism industry (Chen et al., 2020).

### Brand loyalty

In recent years, brand loyalty has been one of the most emphasized concepts, and academics and practitioners are convinced of its relevance (Safeer et al., 2021c; Chen et al., 2022; Zhao et al., 2022). Brand loyalty is defined as a consumer’s evaluations and behavioral intentions of the likelihood of purchasing a particular brand (Safeer et al., 2021c). The primary goal of a firm is to build consumer brand loyalty, which is part of the firm’s strategic planning to achieve long-term competitive advantage (Jin et al., 2013). Similarly, brand loyalty decreases marketing costs and strengthens relationships between consumers and vendors, hence lessening competitors’ threats (Kim et al., 2021). Considering the significance of brand loyalty. This research identified a potential gap in consumer brand loyalty which is a much-debated topic in the current era, particularly in Asia (Lin et al., 2019; Zameer et al., 2019; Safeer et al., 2021c). Thus, the purpose of this study is to examine brand loyalty in the Asian consumer environment.

## Theoretical context and hypotheses development

**FIGURE 1:**
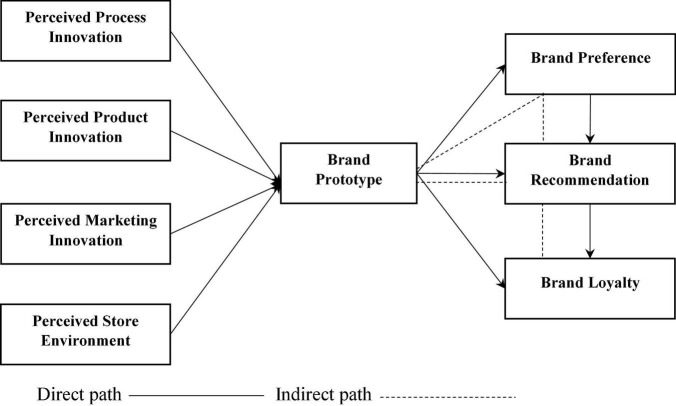
Research model.

This study concentrated on consumers’ perceptions of firms’ innovation activities that may influence consumer brand loyalty via brand prototypes, preferences, and recommendations in the Asian context. Figure 1 shows the proposed model and categorization theory best fit in the current research setting.

### Categorization theory

According to categorization theory, consumers organize objects in their memories according to distinct cognitive schemes, reducing their complexity and improving information processing structure (Rosch and Mervis, 1975). These schemas reveal “cognitive structures of organized prior knowledge, abstracted from experience with specific instances” (Fiske and Linville, 1980, p. 543). When consumers perceive a new object as part of a particular cognitive category, they retrieve previously stored knowledge and transfer the category associations and evaluation components to the new item (Mervis and Rosch, 1981). Categorization theory posits in consumer research that “consumers construct and use categorical representations to classify, interpret, and understand the information they receive” (Loken et al., 2008). The term “category” refers to the information kept in the memory and is later utilized to classify and assign products or services to a consumer category (Han, 2020). In the process of brand categorization, consumers develop brand prototypes according to their perceptions, and these prototypes assist them in product evaluation (Loken, 2005). The brand prototype denotes a consumer’s knowledge of a brand that expands beyond the brand’s structural attributes and is integrated into the consumer’s thought process. Thus, this knowledge drives the entire process of brand selection and evaluation (Cao et al., 2017). Likewise, consumers form clusters and evaluate products in groupings, which is referred to as categorization, and this categorization assists them in examining objects or brands (Gelici-Zeko et al., 2013; Escalas and Bettman, 2017).

### Perceived innovation activities (process, product, marketing, store environment) and brand prototype

Globalization has a significant impact on innovation, and firms now have greater access to worldwide markets. Similarly, the global rivalry has also intensified due to globalization’s growth. As a result, innovation is critical for all organizations, and firms must innovate their products, processes, and marketing activities to survive in global marketplaces (Bloch, 2007). Likewise, consumers now have greater access to information than ever before, and their responses directly impact firms’ innovation activities (Fitzgerald et al., 2020). Considering the focus on firms and consumers, earlier research in Europe and Asia has concentrated primarily on product, process, marketing, and organizational innovation (Nørskov et al., 2015; Gupta et al., 2016; Medrano and Olarte-Pascual, 2016; Aksoy, 2017; Fuentes-Blasco et al., 2017; Ganzer et al., 2017; Na et al., 2019; Quaye and Mensah, 2019; Zhang et al., 2020). However, this research proposed perceived innovation in product, process, marketing, and store environment through consumers’ lenses to better understand consumer brand loyalty in Asia. This research considered process innovation, product innovation, and marketing innovation as defined in the Oslo Manual 2018 (OECD, 2018), while organizational innovation means the process of carrying out tasks in more innovative ways in the organization (Azar and Ciabuschi, 2017). Thus, organizational innovation has no connection to consumers. Therefore, this research ignored organizational innovation and alternatively added perceived store environment from consumers’ perspective (Kumar and Kim, 2014). As a result, this research considered four distinctive types of innovation from consumers’ perspectives, i.e., perceived process, product, and marketing innovation, as well as store environment innovation.

In recent years, firms are targeting consumers with diverse offerings via brand visibility, brand marketing, and store environment (Kumar and Kim, 2014; Shao et al., 2019). Similarly, firms spread information about their process innovation via their own channels or the media, which assists consumers in forming perceptions about company products and services (Lee and Kim, 2013). Thus, based on their experiences and expectations, consumers develop a prototype of a brand in order to evaluate a product. Product innovation assists firms in introducing new or revamping existing products and services. Consumers perceive product innovation about a company’s offerings (Kahn, 2018). Likewise, consumers perceive marketing innovations via information received from the firm’s marketing channels or media (i.e., advertising and promotional strategies) (Gupta et al., 2016). The presence of stores in a market impacts consumer perception of a particular brand since it conveys the brand’s value to consumers (Chuchu et al., 2018). Similarly, store-related operations of the organization, such as processes, products, and marketing strategies, influence consumers’ perceptions of the firm’s innovation activities (Frank et al., 2016). Thus, it indicates that consumers’ perceptions may vary in response to firms’ changing innovation activities, which may impact brand prototypes. Categorization theory reveals that consumers categorize information in their memories based on unique cognitive schemes, thereby lowering their complexity and enhancing information processing structure (Rosch and Mervis, 1975). Thus, firms’ innovation activities, such as process, product, marketing, and store environment, may influence consumers’ perceptions, which may have a favorable effect on brand prototypes (Loken et al., 2008). Therefore, this research hypothesized the following in the Asian context:

H1: Perceived process innovation positively impacts brand prototype.

H2: Perceived product innovation positively impacts brand prototype.

H3: Perceived marketing innovation positively impacts brand prototype.

H4: Perceived store environment positively impacts brand prototype.

### Brand prototype and brand preference, brand recommendation, brand loyalty

Consumer brand knowledge is intimately linked to the brand prototype (Keller, 1993). The brand prototype is the consumer’s perceptions about the uniformity of brands; it comprises the consumer’s basic knowledge and other criteria for assessing the brand (Lianxiong and Huihuang, 2010). Consumers create brand prototypes based on their perceptions throughout the process of brand categorization. These prototypes are then used to evaluate products (Loken, 2005). Prior research has demonstrated the significance of brand prototypes and consumer brand preferences in different consumer settings (Goedertier et al., 2015; Stanton et al., 2016). Previous research developed a framework and argued that destination knowledge (including awareness and image) might impact destination loyalty (Li et al., 2008). Similarly, prior research indicated that consumers’ perceptions of the novelty and intricacy of store (brand) prototype designs affect their brand loyalty in the retail industry (Murray et al., 2017). According to categorization theory, the brand prototype is described as the consumer’s comprehensive knowledge about the brand that extends beyond the brand’s structural attributes and is incorporated into the consumer’s thinking. This knowledge stimulates brand evaluation and the selection process (Cao et al., 2017). Similarly, consumers construct clusters and evaluate related products in groups collectively referred to as categorization (Gelici-Zeko et al., 2013). Consumers can examine cognitive objects by constructing prototypes and categorizing them based on their characteristics (Escalas and Bettman, 2017). In light of all of the above arguments, we can assume the following hypotheses in the Asian context:

H5: Brand prototype positively impacts brand preference.

H6: Brand prototype positively impacts brand recommendation.

H7: Brand prototype positively impacts brand loyalty.

### Brand preference and brand recommendation

Brand preference has traditionally been equated with buying intentions and has been shown to be a significant predictor of purchasing behavior (Tingchi Liu et al., 2014). Past research revealed that the development of brand preference is one sign that consumers are responding positively to firm innovation (Chowdhury and Khare, 2011). Thus, the effectiveness of innovation is contingent upon its ability to change consumer brand preferences. Additionally, a consumer’s familiarity with a particular brand increases their willingness to recommend it to others (Belén del Río et al., 2001). Consumer brand preferences are critical in understanding consumer perceptions in buying products. Thus, brand preference positively influenced customers’ word-of-mouth, which enhanced the likelihood that they would recommend the brands to other consumers in the hospitality industry (Jalilvand et al., 2016). In recent years, brand preference has become highly relevant, and consumer brand preference may favorably influence brand recommendations (Zameer et al., 2019). Therefore, we can assume the following hypotheses:

H8: Brand preference positively impacts brand recommendation.

### Brand recommendation and brand loyalty

Many businesses are concerned about consumer brand loyalty in today’s uncertain environment (Safeer et al., 2021c). As a result, favorable consumer word of mouth and brand recommendations may impact consumer brand loyalty (Gounaris and Stathakopoulos, 2004). Jiménez-Castillo and Sánchez-Fernández (2019) asserted that digital influencers’ brand recommendations drive followers to purchase a specific brand, hence positively affecting their brand loyalty. In addition, other research studies have asserted that consumers’ brand recommendation is a component of brand loyalty that would positively influence consumer behavior in the tourism industry (Chen et al., 2020). According to the categorization theory, consumers’ long-term retention of brand knowledge in their cognitive processes constitutes categorization (Loken et al., 2008). They develop favorable views toward brand recommendations, thereby increasing brand loyalty. This study believes that recommendations of global brands will assist other consumers in developing brand loyalty for global brands. Thus, we can assume:

H9: Brand recommendation positively impacts brand loyalty.

## Materials and methods

### Research approach

The descriptive empirical research was conducted using the online survey method to collect data in accordance with the research objectives. The purpose of descriptive research is to explain a particular population’s assumptions and descriptive statements (Sekaran and Bougie, 2016). Similarly, a survey is described as “a system for collecting information from or about people to describe, compare, or explain their knowledge, attitudes, and behavior” (Sekaran and Bougie, 2016). Prior research revealed that online surveys are the most accessible and cost-effective method for collecting data from the desired population (Rasool et al., 2021). Before developing the formal questionnaire, we conducted focus group discussions to understand consumers’ perceptions of global brands.

### Questionnaire design

After understanding consumers’ familiarization with global brands, this research used several global brands from manufacturing and service industries, including technological brands, i.e., iPhone, Samsung, global restaurants brands, i.e., McDonald, KFC, and media brands, i.e., Netflix and Spotify Music. The global brands were selected based on the nature of their durability and non-durability in order to generalize the findings and implications. Further, the diversified brands were chosen to lessen the specific effects of the brands. We created a standardized questionnaire in the English language. We completed a preliminary questionnaire evaluation with the assistance of two professors and then launched the questionnaire after incorporating their suggestions. Using a well-known Chinese survey website,^1^ an online questionnaire was published to collect data from the intended consumers (see Appendix 1). Using smart computers, it is simple to construct and administer online questionnaire surveys (Sekaran and Bougie, 2016).

### Sampling and data collection

In order to collect data from a mass audience, we released a structured questionnaire on social media platforms, such as WeChat, Facebook, and Whatsapp. We shared the online questionnaire with several social media groups. Using convenience sampling, we received 814 responses from consumers of various Asian countries. Convenience sampling is extensively used in marketing, consumer behavior, and social sciences research (Safeer et al., 2021a,^2022^). According to the research objectives, we eliminated the responses received from non-Asian consumers. After conducting a rigorous data screening process and eliminating many responses (including outliers and straight-lining responses) using SPSS version 25, we considered 571 responses from Asian consumers. We mainly received responses from Chinese, Pakistani, and Indonesian consumers, owing to the fact that all three of these countries were listed in the top ten of the world’s population rankings (Population, 2021). The final data analysis considered 261 responses from China, 191 responses from Pakistan, and 119 responses from Indonesian consumers. The consumer brand selection ratio was 22% for iPhone, 17% for Samsung, 19% for McDonald’s, 18% for KFC, 15% for Netflix, and 9% for Spotify Music. Many researchers suggested that a sample size between 300 to 500 is reflected good, and greater than 500 is considered a very good sample size for data analysis based on covariance-based structural equation modeling (SEM) (Hair et al., 2010). Thus, our sample size follows prior research recommendations and provides sufficient support for data analysis.

### Measures

This research adapted well-established scales.

#### Perceived process innovation

This study adapted the items of perceived process innovation from Lee et al. (2013) and Zameer et al. (2019). Four items were used to measure the construct using a seven-point Likert scale. The items were as (1). This brand’s company places a strong emphasis on research and development, (2). In terms of technology, this brand’s company is a market leader, (3). This brand’s products are made with the most advanced technology, (4). This company frequently uses innovative technology.

#### Perceived product innovation

This study modified the items of perceived product innovation from Lee et al. (2013) and Zameer et al. (2019). Five items were used to measure the construct using a seven-point Likert scale. The items were as (1). This brand’s company offers a variety of product lines, (2). In comparison to its competitors, this brand’s company offers significantly innovative products, (3). This brand’s company is always the first in the industry to introduce new products, (4). In comparison to its competitors, this brand’s company always launches more new products, (5). This brand’s new products are highly innovative.

#### Perceived marketing innovation

This study modified the items of perceived marketing innovation from Im and Workman (2004) and Gupta et al. (2016). Four items were used to measure the construct using a seven-point Likert scale. The items were as (1). Different marketing activities are frequently introduced by the company of this brand and the industry, (2). In comparison to competitors, the brand marketing activities of this company are quite innovative, (3). This brand’s company is constantly using innovative advertising to challenge traditional advertising, (4). This brand’s company takes the initiative in developing new marketing channels.

#### Perceived store environment

This study modified the items of perceived store environment from Kumar and Kim (2014). Four items were used to measure the construct using a seven-point Likert scale. The items were as (1). The staff at this brand’s official store has always been knowledgeable, courteous, and helpful, (2). I like the product selection at this brand’s official store, (3). This brand’s store environment is innovative and appealing, (4). The product adjustments in this brand’s official store are impressive.

#### Brand prototype

This study modified the items of the brand prototype from Keller (2003) and Zameer et al. (2019). Four items were used to measure the construct using a seven-point Likert scale. The items were as (1). This brand has a lot of promotion power, (2). This brand’s business and marketing activities are carried out in various ways, (3). I am aware that this is a well-known brand, (4). This brand provides products that are designed to meet the consumer’s needs.

#### Brand preference

This study modified the items of brand preference from Wang (2013). Three items were used to measure the construct using a seven-point Likert scale. The items were as (1). If other brands are better, it is logical to always select products from this brand, (2). This is the first brand that comes to mind, (3). I will buy this brand’s products next time.

#### Brand recommendation

This study modified the items of brand recommendation from Vigripat and Chan (2007). Two items were used to measure the construct using a seven-point Likert scale. The items were as (1). I think this is a fantastic brand, (2). I will recommend this brand’s products to others.

#### Brand loyalty

This study modified the items of brand loyalty from Nam et al. (2011). Three items were used to measure the construct using a seven-point Likert scale. The items were as (1). Over the next few years, I will stick with my current brand, (2). This is a brand that I recommend to my family and friends, (3). I tell other people positive things about this brand.

## Statistical analysis

Structural equation modeling (SEM) is commonly used to examine the inter-dependent relationships and is the best technique for path analysis and evaluation of model fit. As a result, we employed AMOS version 24 to estimate the SEM using the maximum likelihood approach (Byrne, 2010).

### Preliminary analysis

The data normality test is critical in the initial stages to confirm that the collected data is normalized and appropriate for statistical analysis. A lack of data normalization can impact the validity and reliability of data for multivariate analysis (Hair et al., 2010). To assure data normalization, we utilized multiple strategies to exclude biased data. For example, we examined all responses’ standard deviation (SD) and eliminated the responses with 0 SD, demonstrating that respondents’ responses to all questions were the same. As a result, we eliminated many similar responses. Likewise, we utilized SPSS version 25 to identify and delete several outliers. In addition, we examined the collinearity of the data and found that all average variance extracted (AVE) values were less than 5 and that multicollinearity was not a threat to the data (Byrne, 2010; Hair et al., 2010). We did not find any missing values in the collected data due to fixing restrictions on the online questionnaire. All respondents had to respond to all questions to submit the online questionnaire successfully. In order to assure data normalization, 243 responses were removed. Finally, we considered 571 responses for data analysis.

### Demographic information

According to 571 responses, consumers were 55.52% (317) Male and 44.48% (254) Females. They were 45.71% (261) Chinese, 33.45% (191) Pakistani and 20.84% (119) Indonesian consumers. The consumer demographics information includes their age, education, and monthly income. For example, this research included consumers of various ages: 34.85% (199) were between the ages of 19 – 24 years, 29.77% (170) were between the ages of 25 – 30 years, 17.69% (101) were between the ages of 31 – 35 years, 10.86% (62) were between the ages of 36 – 41 years, and 6.83% (39) were more than 41 years old. Similarly, consumers with varying levels of education participated in this study, including 2.63% (15) with a high school diploma, 45.01% (257) with a bachelor’s degree, 38.18% (218) with a master’s degree, 11.91% (68) with a doctoral degree, and 2.28% (13) consumers were with other professional degrees. Finally, the consumers who participated had a range of monthly incomes, including 21.37% (122) consumers’ with an income of up to USD $1,000, 29.42% (168) consumers’ with an income of between $1,001 and $1,500, 18.39% (105) consumers’ with an income of between $1,501 and $2,000, 12.43% (71) consumers’ with an income of between $2,001 and $2,500, 7.18% (41) consumers’ with an income of between $2,501 and $3,000, and 11.21% (64) consumers’ with an income of more than $3,000 (see Table 1).

**TABLE 1:** Consumers’ demographic information.

Description	Numbers
**Sample size (Consumers**’ **responses)**	**571**
	**Percentage**
China	45.71%
Pakistan	33.45%
Indonesia	20.84%
**Gender**	
Male	55.52%
Female	44.48%
**Age**	
19 – 24	34.85%
25 – 30	29.77%
31 – 35	17.69%
36 – 41	10.86%
More than 41	6.83%
**Education**	
High school	2.63%
Bachelor	45.01%
Master	38.18%
Doctoral	11.91%
Other professional degree	2.28%
**Family income/Month**	
Up to USD $1,000	21.37%
USD $1,001–$1,500	29.42%
USD $1,501–$2,000	18.39%
USD $2,001–$2,500	12.43%
USD $2,501–$3,000	7.18%
More than $3,000	11.21%

### Model measurement evaluation

Before moving to confirmatory factor analysis (CFA), we examined the Cronbach’s Alpha using SPSS version 25 and discovered that all Cronbach’s Alpha values were greater than 0.70, indicating that the scales were sufficiently reliable in the Asian context. Following that, a CFA was executed to evaluate the fitness parameters of the model (Byrne, 2010). Table 2 revealed that all the model indices, such as x^2^/df = 1.61, GFI = 0.94, AGFI = 0.92, CFI = 0.98, NFI = 0.96, SRMR = 0.02, RMSEA = 0.03 and PCLOSE = 1.00 were significant and excellent fit (Hu and Bentler, 1999).

**TABLE 2:** Model fit measures.

Measure	Estimate	Threshold	Interpretation
CMIN	562.53	–	–
DF	349	–	–
CMIN/DF	1.61	Between 1 and 3	Excellent
GFI	0.94	>0.90	Excellent
AGFI	0.92	>0.85	Excellent
CFI	0.98	>0.95	Excellent
NFI	0.96	>0.95	Excellent
SRMR	0.02	<0.08	Excellent
RMSEA	0.03	<0.06	Excellent
PCLOSE	1.00	>0.05	Excellent

CMIN/DF (minimum discrepancy per degree of freedom), GFI (goodness-of-fit index), AGFI (adjusted goodness-of-fit index), CFI (comparative fit index), NFI (non-normed fit index), SRMR (Standardized Root Mean Square Residual), RMSEA (Root Mean Squared Error of Approximation).

This study first assessed the loading values of constructs and observed that all construct indicators values were greater than 0.70 (see Table 3), which satisfied the recommended threshold (Hair et al., 2013). We analyzed the composite reliability (CR) of the constructs and discovered that all CR values were (>0.70) at the threshold level. Similarly, the AVE values were greater than 0.50 and satisfied the recommended criterion (Hair et al., 2013). We checked the discriminant validity by following the HTMT approach and discovered that all HTMT values (see Table 4) were (<0.90) in accordance with the recommended threshold (Henseler et al., 2015). As a result, we established discriminant validity.

**TABLE 3:** Constructs loading, composite reliability (CR), and average variance extracted (AVE).

Code	Construct	Loading value	CR	AVE
**Perceived process innovation (PPSI)**			0.92	0.73
PPSI1	This brand’s company places a strong emphasis on research and development.	0.85		
PPSI2	In terms of technology, this brand’s company is a market leader.	0.84		
PPSI3	This brand’s products are made with the most advanced technology.	0.87		
PPSI4	This company frequently uses innovative technology	0.86		
**Perceived product innovation (PPTI)**			0.92	0.69
PPTI1	This brand’s company offers a variety of product lines.	0.81		
PPTI2	In comparison to its competitors, this brand’s company offers significantly innovative products.	0.83		
PPTI3	This brand’s company is always the first in the industry to introduce new products.	0.84		
PPTI4	In comparison to its competitors, this brand’s company always launches more new products.	0.83		
PPTI5	This brand’s new products are highly innovative.	0.83		
**Perceived marketing innovation (PMI)**			0.89	0.68
PMI1	Different marketing activities are frequently introduced by the company of this brand and the industry.	0.80		
PMI2	In comparison to competitors, the brand marketing activities of this company are quite innovative.	0.85		
PMI3	This brand’s company is constantly using innovative advertising to challenge traditional advertising.	0.82		
PMI4	This brand’s company takes the initiative in developing new marketing channels.	0.83		
**Perceived Store Environment (PSE)**			0.92	0.75
PSE1	The staff at this brand’s official store has always been knowledgeable, courteous, and helpful.	0.86		
PSE2	I like the product selection at this brand’s official store.	0.86		
PSE3	This brand’s store environment is innovative and appealing.	0.87		
PSE4	The product adjustments in this brand’s official store are impressive.	0.87		
**Brand prototype (BP)**			0.92	0.75
BP1	This brand has a lot of promotion power.	0.87		
BP2	This brand’s business and marketing activities are carried out in various ways.	0.85		
BP3	I am aware that this is a well-known brand.	0.88		
BP4	This brand provides products that are designed to meet the consumer’s needs.	0.86		
**Brand preference (BPF)**			0.89	0.74
BPF1	If other brands are better, it is logical to always select products from this brand.	0.83		
BPF2	This is the first brand that comes to mind.	0.86		
BPF3	I will buy this brand’s products next time.	0.88		
**Brand recommendation (BR)**			0.87	0.78
BR1	I think this is a fantastic brand.	0.89		
BR2	I will recommend this brand’s products to others.	0.87		
**Brand loyalty (BL)**			0.86	0.68
BL1	Over the next few years, I will stick with my current brand.	0.81		
BL2	This is a brand that I recommend to my family and friends.	0.82		
BL3	I tell other people positive things about this brand	0.84		

**TABLE 4:** Heterotrait monotrait analysis.

	1	2	3	4	5	6	7	8
1. PPSI								
2. PMI	0.75							
3. PPTI	0.77	0.80						
4. PSE	0.73	0.79	0.71					
5. BP	0.79	0.74	0.76	0.73				
6. BPF	0.79	0.66	0.71	0.68	0.73			
7. BL	0.73	0.68	0.73	0.68	0.76	0.78		
8. BR	0.82	0.74	0.76	0.77	0.83	0.83	0.81	

### Structural model evaluation

Following the measurement model evaluation, we assessed the structural model using the structural equation modeling technique with bootstrapping 5000 subsamples at a 95% confidence interval (Byrne, 2010). We discovered that the structural model was significant with an excellent fit, as indicated by the following values: x^2^/df = 2.04, GFI = 0.92, AGFI = 0.90, CFI = 0.97, NFI = 0.95, RMSEA = 0.04. Table 5 summarizes the outcomes of the structural model’s proposed hypotheses. We discovered that perceived process innovation and perceived product innovation significantly impacted brand prototypes. Thus, H1 and H2 were supported. However, we found that perceived marketing innovation had no impact on the brand prototype, as PMI==>BP (β = 0.080, *p* = 0.176). Therefore, H3 was not supported. Further, we found that perceived store environment positively impacted brand prototype. Thus, H4 was also supported. Similarly, brand prototypes significantly affected brand preference, brand recommendation, and brand loyalty in Asia. Therefore, H5 – H7 were supported. Likewise, brand preference positively affected brand recommendation. As a result, H8 was supported. Finally, brand recommendation positively influenced consumer brand loyalty in Asia. Hence, H9 was also supported.

**TABLE 5:** Hypotheses testing.

Hyp.	Structural relationships			Estimate	S.E.	C.R.	p-value	Support
H1	PPSI	===>	BP	0.42	0.05	8.09	***	Yes
H2	PPTI	===>	BP	0.25	0.06	4.74	***	Yes
H3	PMI	===>	BP	0.08	0.06	1.35	0.18	No
H4	PSE	===>	BP	0.22	0.05	4.37	***	Yes
H5	BP	===>	BPF	0.78	0.04	18.84	***	Yes
H6	BP	===>	BR	0.53	0.05	10.82	***	Yes
H7	BP	===>	BL	0.28	0.08	3.40	***	Yes
H8	BPF	===>	BR	0.44	0.05	9.02	***	Yes
H9	BR	===>	BL	0.59	0.08	6.78	***	Yes

C.R. (Critical Ratio) > 3.29, ***p < 0.001; S.E. (Standard Error); Hyp. (Hypothesis).

**FIGURE 2:**
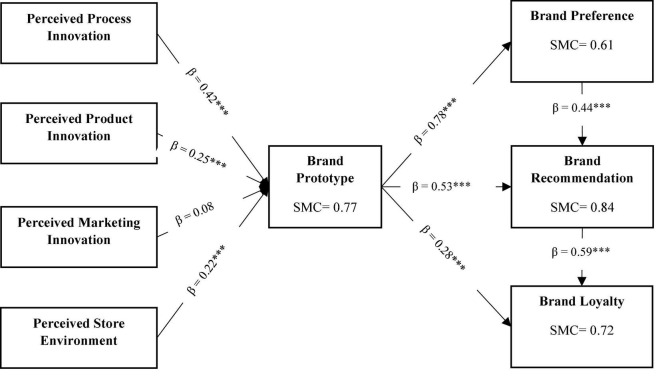
Structural model relationships and squared multiple correlations (SMC).

The squared multiple correlation (SMC) values indicate the percentage of variance explained by the variable’s predictors (Byrne, 2010). Figure 2 illustrated the SMC values in which exogenous variables accounted for 77.40% of explained variance to endogenous variables, i.e., brand prototype. Similarly, other exogenous variables accounted for the explained variance of 61.10% to brand preference, 84.10% to brand recommendation, and 71.60% to brand loyalty. As a result, it was demonstrated that the proposed model possessed excellent explanatory power.

Additionally, we conducted mediation analysis using the approaches advocated by various authors (Baron and Kenny, 1986; Preacher and Hayes, 2008). We evaluated the direct and indirect relationships and identified partial mediation to examine the mediation (see Table 6).

**TABLE 6:** Mediation results.

Structural relationships					Indirect	Indirect p-value	Direct	Mediation
BP	===>	BPF	===>	BR	0.35	***	Significant	Partial
BP	===>	BR	===>	BL	0.51	***	Significant	Partial

Significance level: ^***^p < 0.001.

## Discussions

This study uncovered several intriguing insights on consumer perceptions of firms’ innovation activities in the Asian context. First, this study discovered that consumers’ perceptions of the process and product innovation (H1–H2) had a substantial effect on brand prototypes in Asia. These findings corroborate prior research by Zameer et al. (2019), who discovered that product and process innovation significantly affected consumers’ prototypes by increasing their knowledge. As a result, process and product innovation positively influence consumers’ brand evaluation. Additionally, perceived product innovation significantly impacted the brand prototype, which assisted consumers in recognizing brands in the United States (Keller et al., 2011). Companies use their own channels or the media to distribute information about their process innovation, which helps consumers develop their own perceptions about the company’s products and services (Lee and Kim, 2013; Kahn, 2018). Thus, our findings corroborate and reinforce that consumers’ perceptions of firms’ processes and product innovations benefit consumers in generating positive brand prototypes and increasing consumers’ knowledge of brands.

Second, this study discovered (H3) that consumers’ perceptions of marketing innovation did not affect the brand prototype. These findings contrast with previous studies indicating that consumers’ views of marketing activities had a favorable effect on brand prototypes in China and Turkey (Akgun et al., 2017; Zameer et al., 2019). There are few studies published on this topic. As a result, we recommend additional research to validate consumers’ perceptions of firms’ innovative marketing initiatives in the Asian environment. H4 discovered that consumers’ perceptions of the store environment had a favorable effect on the brand prototype, which resulted in an increase in consumer brand knowledge. The findings are consistent with earlier research by Kumar and Kim (2014). They discovered that store environment factors such as design, ambient, and social influence positively affect consumers’ evaluations, aid in increasing their knowledge about firm products and positively influence their behavior toward store brands in the United States. Thus, the findings revealed that the store environment enables firms to increase consumer brand knowledge that favorably affects their behavior, particularly in Asia.

H5–H7 indicated that brand prototypes positively influenced consumers’ brand preferences, brand recommendations, and brand loyalty in Asia. These findings corroborate prior studies, indicating that brand knowledge (prototype) contributes to consumers’ favorable perceptions of their brand preference, recommendation, and loyalty in the United States, China, and Spain (Palumbo and Herbig, 2000; Del Rio et al., 2001; Zameer et al., 2019). Thus, this research established that prototypes assist consumers in increasing their knowledge about brands, which has a favorable effect on the development of their preferences, recommendations to other consumers, and brand loyalty in Asia. H8 discovered that brand preferences positively influence their brand recommendations, and similarly, H9 revealed that brand recommendation contributes to brand loyalty development. Prior studies validate our findings, explaining that consumers’ brand knowledge (prototypes) helps them in developing their preferences toward global brands. Similarly, consumers’ perceptions and brand preferences positively impact their willingness to recommend a specific brand to other consumers, and brand recommendations significantly influence consumer brand loyalty in Europe (Greece and United Kingdom) (Jamal and Goode, 2001; Gounaris and Stathakopoulos, 2004). As a result, our findings validated that Asian consumers have a similar thinking pattern in that their brand preference leads to recommendations, which in turn positively influences their loyalty toward global brands.

## Conclusion

This study examined consumer perceptions of firm innovation activities and their impact on the brand prototype, which leads to brand preference, recommendation, and loyalty of global brands in Asian environments, focusing on China, Pakistan, and Indonesia. This study concluded that Asian (Chinese, Pakistani, and Indonesian) consumers have favorable perceptions of firms’ innovation activities (including process, product, and store environment innovation), which influences their ability to develop brand prototypes to increase consumers’ knowledge of global brands. Similarly, brand knowledge (prototype) supports the development of global brand preferences, brand recommendations, and brand loyalty among Asian consumers. As a result of their positive preference for global brands, consumers are most likely to recommend global brands to other consumers, such as their family, friends, and relatives. Consequently, consumers’ recommendations of global brands favorably influence their brand loyalty. The findings revealed that global corporations should invest in their firm’s innovation activities by improving their processes, products, and store environment, which would have a favorable effect on brand prototypes in terms of increasing consumer knowledge about global brands. Similarly, their brand knowledge will drive them to increase their global brand preferences and recommendations, as well as nurture their global brand loyalty in Asia. Likewise, consumer global brand preferences and brand recommendations assist companies in fostering consumer loyalty, thereby strengthening their business sustainability in Asia. Asia is a lucrative market segment for global brands (Safeer et al., 2021a). Therefore, this study may assist multinational corporations in increasing their business volumes and market shares in Asian markets.

### Theoretical contributions

This study makes novel contributions to categorization theory. First, this study validated the perceived innovation activities and brand-related scales (brand prototype, brand preference, brand recommendation, and brand loyalty) in the Asian environment. Second, this study contributes to our understanding of consumer perceptions of innovation activities and their effects on brand prototypes in an Asian setting by demonstrating that perceived innovation activities (process, product, and store environment) increase consumer brand knowledge. Categorization theory states that individual cognitive structures of arranged existing knowledge derived from experience with particular situations (Fiske and Linville, 1980). Likewise, when consumers see a new object referring to a specific cognitive category, they access previously learned information and transfer category connections and judgment factors to the new object (Mervis and Rosch, 1981). Thus, using cognitive structure, consumers evaluate the innovation actions of corporations, thereby increasing their brand knowledge.

Third, this study discovered that brand prototypes significantly strengthen brand preference, brand recommendation, and brand loyalty in Asia. The theory states that throughout the brand categorization process, consumers form brand prototypes depending on their perceptions, which are then used to appraise items (Loken, 2005). Consequently, consumer brand knowledge stimulates consumers’ brand preference, recommendation, and loyalty among Asian consumers toward global brands. Finally, this study found that consumer brand preference leads to the brand recommendation, which strengthens brand loyalty among Asian consumers. Prior research revealed that brand preferences can increase consumers’ willingness to recommend a brand in Iran (Jalilvand et al., 2016) and that consumer recommendations can increase brand loyalty in Greek (Gounaris and Stathakopoulos, 2004). Thus, our findings provide a novel contribution to Asian environments, particularly China, Pakistan, and Indonesia.

### Managerial recommendations

This study provides several recommendations to global marketers and brand managers. First, this research discovered that global managers must understand consumers’ perceptions before planning innovation activities, such as innovation in processes, products, and store environments, which significantly influence consumers’ perceptions of brand prototypes by increasing their knowledge about brands. Therefore, managers should prioritize strengthening their innovation initiatives by understanding Asian consumer perceptions. Second, this study found that brand prototypes positively influence brand preference, brand recommendation, and brand loyalty in Asia. Global managers need to design brand prototypes to improve consumer brand knowledge, which may drive them to increase their brand preference, recommendations, and loyalty toward global brands. Global managers can increase brand knowledge among Asian consumers by implementing various social media and other media campaigns. Similarly, such consumer brand knowledge can assist managers in developing and implementing brand positioning strategies, eventually enhancing brand preference, brand recommendation, and brand loyalty among Asian consumers. Finally, this study found that brand preference leads to brand recommendation, which influences brand loyalty positively in Asia. Global managers may strengthen brand prototypes (knowledge) to increase brand preference among Asian consumers, thereby encouraging them to recommend the brand to others. Thus, brand recommendations will increase brand loyalty among Asian consumers. As a result, increasing consumer brand loyalty in Asia may assist corporations in achieving greater business sustainability.

### Limitations and future research scope

This research has some limitations. First, according to the OSLO manual, this study was focused on a few innovation activities in the Asian environment, particularly in China, Pakistan, and Indonesia. Future studies may incorporate further innovation-related activities in order to better comprehend consumer behavior by applying cross-cultural comparative research, such as comparing Asia to Europe or the United States. Second, this study analyzed data from three Asian countries (China, Pakistan, and Indonesia). Future researchers may collect further data from other Asian countries on a larger scale in order to generalize the findings to the Asian environment. Third, this study was focused on a limited number of product categories and brands. Future research may increase the product categories and brands to generate more potential contributions in Asian settings. Fourth, due to the complexity of the proposed model, this study did not investigate the impact of innovation activities (i.e., process, product, marketing, and store environment) on brand preference, brand recommendation, and brand loyalty. Future research may investigate the impact of innovation activities (i.e., process, product, marketing, and store environment) on brand preference, brand recommendation, and brand loyalty to reveal new insights in Asia. Finally, this study used a brand prototype as a mediator. Future research may use the mediating function of brand experience to uncover insightful findings in Asia.

## Data availability statement

The raw data supporting the conclusions of this article will be made available by the authors, without undue reservation.

## Author contributions

LY recognized the research gap, managed the introduction, created a theoretical model, and developed hypotheses. MK worked on methodology, performed analysis, and interpreted the results. AAS collected the data and worked on results, discussions, theoretical contributions, managerial recommendations, conclusion, limitations, and future research scope of this study. All authors read and approved the final manuscript.

## Conflict of interest

The authors declare that the research was conducted in the absence of any commercial or financial relationships that could be construed as a potential conflict of interest.

## Publisher’s note

All claims expressed in this article are solely those of the authors and do not necessarily represent those of their affiliated organizations, or those of the publisher, the editors and the reviewers. Any product that may be evaluated in this article, or claim that may be made by its manufacturer, is not guaranteed or endorsed by the publisher.

## Footnotes

1
https://www.wjx.cn

## Appendix

### Appendix 1 Questionnaire

**Section 2: Please SELECT ONE BRAND in the following list and fill the questionnaire accordingly.**

According to your brand knowledge/experience, please select **one most familiar global brands** in the following brands and fill the questionnaire accordingly.

1. iPhone 2. Samsung 3. McDonald 4. KFC 5. Netflix 6. Spotify Music

Please review the following questions and answer with your best understanding by choosing a score by using tick mark (**√**) (1-7) according to the given seven points rating scale mentioned (1 = Strongly Disagree, 2 = Disagree, 3 = Somewhat Disagree, 4 = Undecided, 5 = Somewhat Agree, 6 = Agree, 7 = Strongly Agree).

**Table:**

Code	Construct	Strongly Disagree	Disagree	Somewhat Disagree	Undecided	Somewhat Agree	Agree	Strongly Agree
**Perceived Process Innovation (PPSI)**		1	2	3	4	5	6	7
PPSI1	This brand’s company places a strong emphasis on research and development.	1	2	3	4	5	6	7
PPSI2	In terms of technology, this brand’s company is a market leader.	1	2	3	4	5	6	7
PPSI3	This brand’s products are made with the most advanced technology.	1	2	3	4	5	6	7
PPSI4	This company frequently uses innovative technology	1	2	3	4	5	6	7
**Perceived Product Innovation (PPTI)**		1	2	3	4	5	6	7
PPTI1	This brand’s company offers a variety of product lines.	1	2	3	4	5	6	7
PPTI2	In comparison to its competitors, this brand’s company offers significantly innovative products.	1	2	3	4	5	6	7
PPTI3	This brand’s company is always the first in the industry to introduce new products.	1	2	3	4	5	6	7
PPTI4	In comparison to its competitors, this brand’s company always launches more new products.	1	2	3	4	5	6	7
PPTI5	This brand’s new products are highly innovative.	1	2	3	4	5	6	7
**Perceived Marketing Innovation (PMI)**		1	2	3	4	5	6	7
PMI1	Different marketing activities are frequently introduced by the company of this brand and the industry.	1	2	3	4	5	6	7
PMI2	In comparison to competitors, the brand marketing activities of this company are quite innovative.	1	2	3	4	5	6	7
PMI3	This brand’s company is constantly using innovative advertising to challenge traditional advertising.	1	2	3	4	5	6	7
PMI4	This brand’s company takes the initiative in developing new marketing channels.	1	2	3	4	5	6	7
**Perceived Store Environment (PSE)**		1	2	3	4	5	6	7
PSE1	The staff at this brand’s official store has always been knowledgeable, courteous, and helpful.	1	2	3	4	5	6	7
PSE2	I like the product selection at this brand’s official store.	1	2	3	4	5	6	7
PSE3	This brand’s store environment is innovative and appealing.	1	2	3	4	5	6	7
PSE4	The product adjustments in this brand’s official store are impressive.	1	2	3	4	5	6	7
**Brand Prototype (BP)**		1	2	3	4	5	6	7
BP1	This brand has a lot of promotion power.	1	2	3	4	5	6	7
BP2	This brand’s business and marketing activities are carried out in various ways.	1	2	3	4	5	6	7
BP3	I am aware that this is a well-known brand.	1	2	3	4	5	6	7
BP4	This brand provides products that are designed to meet the consumer’s needs.	1	2	3	4	5	6	7
**Brand Preference (BPF)**		1	2	3	4	5	6	7
BPF1	If other brands are better, it is logical to always select products from this brand.	1	2	3	4	5	6	7
BPF2	This is the first brand that comes to mind.	1	2	3	4	5	6	7
BPF3	I will buy this brand’s products next time.	1	2	3	4	5	6	7
**Brand Recommendation (BR)**		1	2	3	4	5	6	7
BR1	I think this is a fantastic brand.	1	2	3	4	5	6	7
BR2	I will recommend this brand’s products to others.	1	2	3	4	5	6	7
**Brand Loyalty (BL)**		1	2	3	4	5	6	7
BL1	Over the next few years, I will stick with my current brand.	1	2	3	4	5	6	7
BL2	This is a brand that I recommend to my family and friends.	1	2	3	4	5	6	7
BL3	I tell other people positive things about this brand	1	2	3	4	5	6	7
